# Optimizing Pain Relief and Range of Motion in Unilateral Cervical Radiculopathy: A Study on Neural Tissue Mobilization and Cervical Stabilization Exercises

**DOI:** 10.7759/cureus.65646

**Published:** 2024-07-29

**Authors:** Vaibhav Agarwal, Amit Goel, Abhay Srivastava, Praveen Rawat, Rajender Singh

**Affiliations:** 1 Physiotherapy, Himalayan Institute of Medical Sciences, Swami Rama Himalayan University, Dehradun, IND; 2 Physiotherapy, Jyotirao Phule Subharti College of Physiotherapy, Meerut, IND; 3 Community Medicine, Himalayan Institute of Medical Sciences, Swami Rama Himalayan University, Dehradun, IND; 4 Microbiology, Himalayan Institute of Medical Sciences, Swami Rama Himalayan University, Dehradun, IND

**Keywords:** range of motion, neck pain, cervical stabilization exercises, neural mobilization, cervical radiculopathy

## Abstract

Background

This study aimed to analyze the combined effect of neural mobilization along with cervical stabilization exercises on pain and cervical range of motion in unilateral cervical radiculopathy patients.

Methodology

A total of 30 patients aged 30-45 years with unilateral cervical radiculopathy were randomly divided into the following two groups: experimental (n = 15) and control (n = 15). The experimental group received neural mobilization along with cervical stabilization exercises, while the control group received conventional treatment. Outcome measures included pain intensity measured on a visual analog scale (VAS), functional status of the neck measured by the Neck Disability Index (NDI), and cervical range of motion measured by a goniometer. All measures were taken before treatment, after treatment, and at the one-week follow-up.

Results

The results showed statistically significant positive improvements in VAS, NDI score, and cervical range of motion in unilateral cervical radiculopathy subjects of the experimental group.

Conclusions

Neural mobilization combined with cervical stabilization exercises led to significant improvements in pain, functional status, and cervical range of motion in patients with unilateral cervical radiculopathy compared to conventional treatment.

## Introduction

Cervical radiculopathy is characterized by radicular pain in the arm and neck, numbness, sensory deficits, and motor deficits due to nerve compression in the spine. The most common causes are disc herniation and spondylosis [1]. Cervical radiculopathy has an average incidence rate of 83 per 100,000 for the general population, with a higher prevalence in the fifth decade of life (203 per 100,000) [2]. Forward head posture is often seen in patients with neck problems, leading to increased load on the posterior cervical structures and weakness of the deep cervical muscles. Cervical stabilization exercises can improve cervical stabilization, mobility, vertebral alignment, and sensorimotor function. Neural mobilization aims to restore the plasticity of the nervous system and improve the neurophysiological and neuromechanical functions of the peripheral nervous system. Transcutaneous electrical nerve stimulation (TENS) is a physiotherapy technique using a small device that sends electrical impulses via skin electrodes to stimulate nerves and reduce pain perception. It effectively relieves acute and chronic pain, reduces inflammation, improves flexibility and muscle strength, and enhances circulation. Its benefits include being non-invasive, portable, with minimal side effects, and cost-effective. TENS is used to treat musculoskeletal injuries, chronic conditions such as arthritis and fibromyalgia, neuropathic pain such as diabetic neuropathy, and postoperative pain. TENS along with stretching exercises have been used to reduce the pain. This study aimed to examine the combined effects of neural tissue mobilization and cervical stabilization exercises against conventional physiotherapy in unilateral cervical radiculopathy patients.

## Materials and methods

This experimental study recruited 30 patients with unilateral cervical radiculopathy aged between 30 and 45 years from Himalayan Hospital, Swami Rama Himalayan University, Dehradun, India. Before treatment, all patients underwent detailed screening based on the inclusion and exclusion criteria.

Inclusion criteria

The following inclusion criteria were considered: both male and female patients with forward head posture (craniovertebral angle <45°) aged between 30 and 45 years; unilateral radicular pain in the upper limb for less than three months; the cervical range of motion <45° of extension, <25° ipsilateral lateral flexion, and <60° ipsilateral of rotation; and a positive Spurling’s test, distraction test, and median nerve tension test.

Exclusion criteria

The following exclusion criteria were considered: orthopedic or neurological conditions of the cervical spine and shoulder joint; hypermobility of the cervical spine; malignancy; vertebral basilar artery insufficiency; a history of cervical surgeries in the last 10 years; bilateral cervical radiculopathies; and pregnancy.

Patients were then randomly assigned to either the experimental group (n = 15) or the control group (n = 15). The lottery method was used for random allocation.

The experimental group received a novel intervention combining neural mobilization techniques followed by cervical stabilization exercises. Mobilization technique doses were 15-20 repetitions in one session with one minute of rest. Cervical static and dynamic openers were given in the mobilization technique. In the stabilization exercise, the dose was 10 repetitions per session. The stabilization exercise was cervical dynamic isometric, shoulder shrugs, shoulder roll, chest flies, and chin tuck exercises.

Neural mobilization

Neural mobilization was performed using specific movements of the affected arm and neck to enhance nerve gliding and reduce compression. The procedure involved the following steps: (1) Initial positioning: The patient was positioned comfortably, ensuring proper support for the neck and arm. (2) Gentle movements: Gentle, controlled movements were initiated, focusing on the affected arm and neck. Movements included shoulder abduction, elbow extension, and wrist flexion/extension. (3) Progressive range of motion: The range of motion was gradually increased as tolerated by the patient, ensuring no exacerbation of symptoms. (4) Monitoring and adjustments: Continuous monitoring of the patient’s response was conducted, with adjustments made to the movements as necessary to ensure optimal nerve gliding without discomfort.

Cervical stabilization exercises

Cervical stabilization exercises were implemented to strengthen and coordinate the neck muscles, enhancing core support. The procedure involved the following steps: (1) Initial assessment: An initial assessment of neck muscle strength and coordination was performed. (2) Isometric contractions: The exercises began with isometric contractions, where the patient was instructed to tense the neck muscles without producing any movement. This included holding positions such as chin tucks and side holds for specified durations. (3) Dynamic exercises: Progression to dynamic exercises was made, incorporating controlled neck movements such as flexion, extension, lateral bending, and rotation. (4) Core integration: Advanced exercises integrated core muscle engagement, emphasizing coordination between the neck and trunk muscles to improve overall stability. (5) Repetition and progression: Exercises were repeated for a specified number of sets and repetitions, with gradual progression in intensity and complexity based on the patient’s tolerance and improvement.

Both neural mobilization and cervical stabilization exercises were conducted under the supervision of a qualified physiotherapist, with regular assessments to monitor progress and adjust the treatment plan accordingly.

The control group received conventional treatment, serving as a benchmark for comparison. This included (1) TENS: High-frequency (90-130 Hz) TENS was applied to reduce pain. (2) Stretching exercises: Stretching exercises were performed to improve the flexibility of the neck, shoulder, and scapular muscles.

Pain intensity was assessed using the visual analog scale (VAS), functional status of the neck was measured using the Neck Disability Index (NDI), and cervical range of motion was measured using a goniometer. These measurements were taken before treatment, after treatment, and at the one-week follow-up session.

Statistical analysis

The data collected were analyzed using SPSS Version 20 (IBM Corp., Armonk, NY, USA). This analysis compared the changes in pain intensity, neck function, and cervical range of motion between the experimental and control groups. Statistical significance was determined at a p-value of less than 0.05, indicating a statistically relevant difference between the two treatment approaches.

## Results

The results of this study provide valuable insights into the effectiveness of combining neural tissue mobilization and cervical stabilization exercises in the management of unilateral cervical radiculopathy. By analyzing the outcomes of pain intensity, functional status, and cervical range of motion, this study sheds light on the potential benefits of these interventions for patients with this condition. The findings suggest that a multimodal approach targeting both neural and musculoskeletal components may offer significant improvements in patient outcomes compared to conventional treatments. These results underscore the importance of further research in this area to refine treatment protocols and optimize outcomes for patients with cervical radiculopathy. Normality of the data was assessed in both the control and experimental groups, each comprising 15 individuals. Given the small sample size, Shapiro-Wilk tests were employed to evaluate the distribution of the data, ensuring statistical assumptions were met for subsequent analyses.

We employed the independent t-test to compare the results between the control and experimental groups, each comprising 15 individuals. This statistical analysis was chosen based on the normal distribution of our data and adherence to parametric assumptions, ensuring a robust comparison of outcomes.

The VAS scores for pain intensity were measured before and after the intervention in both the experimental and control groups (Table 1). The mean VAS score in the experimental group decreased significantly from 7.33 ± 0.62 to 0.67 ± 0.62 (p < 0.001), indicating a substantial reduction in pain intensity. In contrast, the mean VAS score in the control group also decreased from 6.93 ± 0.70 to 3.27 ± 0.59 (p < 0.001), but the reduction was not as pronounced as in the experimental group (Table 1).

**Table 1:** Comparison of visual analog scale scores between the experimental and control groups using the independent t-test.

Group	Pre-treatment (mean ± SD)	Post-treatment (mean ± SD)	P-value
Experimental	7.33 ± 0.62	0.67 ± 0.62	<0.001
Control	6.93 ± 0.70	3.27 ± 0.60	<0.001

The craniovertebral angle, which reflects the degree of forward head posture, was measured before and after the intervention in both groups. In the experimental group, the mean craniovertebral angle improved significantly from 26.53 ± 2.20 to 42.27 ± 1.16 degrees (p < 0.001), indicating a reduction in forward head posture. Similarly, the control group also showed improvement, with the mean craniovertebral angle changing from 25.27 ± 2.02 to 14.07 ± 0.88 degrees (p < 0.001), but the improvement was greater in the experimental group. Forward head posture is a common postural deviation where the head is positioned anterior to the vertical line through the body and center of gravity. This posture can lead to various musculoskeletal issues, including alterations in neurodynamics and changes in the costovertebral angle (Table 2).

**Table 2:** Comparison of the craniovertebral angle between the experimental and control groups using the independent t-test.

Group	Pre-treatment (mean ± SD)	Post-treatment (mean ± SD)	P-value
Experimental	26.53 ± 2.20	42.27 ± 1.16	<0.001
Control	25.27 ± 2.02	14.07 ± 0.88	<0.001

The NDI scores, assessing functional status concerning neck pain and disability, were compared between the experimental and control groups. In the experimental group, the mean NDI score improved significantly from 36.67 ± 2.13 to 2.27 ± 1.16 (p < 0.001), indicating better functional outcomes. Similarly, the control group also showed improvement, with the mean NDI score reducing from 36.73 ± 1.75 to 14.07 ± 0.88 (p < 0.001), but the improvement was more pronounced in the experimental group (Table 3).

**Table 3:** Comparison of Neck Disability Index scores between the experimental and control groups using the independent t-test.

Group	Pre-treatment (mean ± SD)	Post-treatment (mean ± SD)	P-value
Experimental	36.67 ± 2.13	2.27 ± 1.16	<0.001
Control	36.73 ± 1.75	14.07 ± 0.88	<0.001

The range of ipsilateral cervical lateral rotation was measured before and after the intervention in both groups. In the experimental group, the mean range improved significantly from 52.93 ± 2.05 to 78.13 ± 1.41 degrees (p < 0.001), indicating an increase in the cervical range of motion. Similarly, the control group also showed improvement, with the mean range changing from 53.87 ± 1.77 to 60.87 ± 1.64 degrees (p < 0.001), but the improvement was greater in the experimental group (Table 4).

**Table 4:** Comparison of the ipsilateral cervical lateral rotation range between the experimental and control groups using the independent t-test.

Group	Pre-treatment (mean ± SD)	Post-treatment (mean ± SD)	P-value
Experimental	52.93 ± 2.05	78.13 ± 1.41	<0.001
Control	53.87 ± 1.77	60.87 ± 1.64	<0.001

The angle of the cervical extension was measured before and after the intervention in both groups. In the experimental group, the mean angle improved significantly from 40.67 ± 2.87 to 77.93 ± 2.05 degrees (p < 0.001), indicating an increase in the cervical range of motion for extension. Similarly, the control group also showed improvement, with the mean angle changing from 41.40 ± 1.45 to 67.33 ± 1.80 degrees (p < 0.001), but the improvement was more pronounced in the experimental group (Table 5).

**Table 5:** Comparison of the cervical extension angle between the experimental and control groups using the independent t-test.

Group	Pre-treatment (mean ± SD)	Post-treatment (mean ± SD)	P-value
Experimental	40.67 ± 2.87	77.93 ± 2.05	<0.001
Control	41.40 ± 1.45	67.33 ± 1.80	<0.001

The range of ipsilateral lateral flexion was measured before and after the intervention in both groups. In the experimental group, the mean range improved significantly from 21.93 ± 1.39 to 43.93 ± 1.03 degrees (p < 0.001), indicating an increase in the cervical range of motion for lateral flexion. Similarly, the control group also showed improvement, with the mean range changing from 22.07 ± 1.34 to 41.27 ± 0.70 degrees (p < 0.001), but the improvement was greater in the experimental group (Table 6).

**Table 6:** Comparison of lateral flexion range in the experimental and control groups using the independent t-test.

Study group	Pre-treatment (mean ± SD)	Post-treatment (mean ± SD)	Follow-up (mean ± SD)	P-value
Experimental	21.93 ± 1.39	42.07 ± 1.16	43.93 ± 1.03	<0.001
Control	22.07 ± 1.34	37.33 ± 2.13	41.27 ± 0.70	<0.001

## Discussion

This study aimed to investigate the combined effects of neural tissue mobilization and cervical stabilization exercises on pain and cervical range of motion in patients with unilateral cervical radiculopathy. The results of this study indicate that the combination of these interventions led to significant improvements in pain intensity, functional status, and cervical range of motion compared to conventional treatment.

The findings regarding pain intensity, as measured by the VAS, showed a substantial reduction in pain in both the experimental and control groups. However, the reduction was more pronounced in the experimental group, which received neural mobilization combined with cervical stabilization exercises, showing similar results. This suggests that these interventions may be more effective in reducing pain compared to conventional treatment alone.

The improvement in functional status, as assessed by the NDI, was also more significant in the experimental group. This indicates that the combination of neural mobilization and cervical stabilization exercises may lead to better functional outcomes and a reduced impact of neck pain on daily activities.

The results indicate a notable enhancement in cervical range of motion in both groups, with the experimental group showing even greater improvements. This implies that the interventions employed could be valuable in enhancing cervical mobility and alleviating stiffness among individuals with cervical radiculopathy.

These findings align with earlier research demonstrating the efficacy of neural mobilization and cervical stabilization exercises in mitigating pain and enhancing functional outcomes in patients dealing with neck pain and cervical radiculopathy. For instance, a study by Kim et al. [3] found that neural mobilization with cervical traction was effective in improving VAS scores in cervical radiculopathy patients. The long-term effects of neurodynamics over traction on cervical radiculopathy are due to sustained pain relief which persists for months and even for years, improved mobility and flexibility, increased function without exacerbating symptoms, reduced numbness and tingling, prevention of further damage, and enhanced overall mood, well-being, and sleep quality. Neurodynamics has reduced dependency on pain medication and prevented the development of chronic pain. Similarly, Basson et al. [4] found that neural mobilization was effective in improving VAS scores in cervical radiculopathy patients.

The study by Srinivasulu and Chunduri [5] compared the effectiveness of neural mobilization and Mulligan mobilization in the management of cervical radiculopathy. The study found that neural mobilization was more effective in reducing neck disability function compared to Mulligan mobilization. The effects include enhanced neural sliding, which improves nerve mobility and reduces adhesions. It increases joint range of motion and reduces stiffness. It decreases pain intensity and distribution and enhances proprioception. Neurodynamics can aid in nerve recovery and regeneration, and improve muscle tone, strength, and coordination. It may also minimize scar tissue development after injury. This suggests that neural mobilization may be a valuable intervention for improving outcomes in patients with cervical radiculopathy.

In the study by Kuo et al. [6], the researchers examined how stabilization exercise training impacts pain levels and quality of life among individuals with cervical radiculopathy. They discovered that such training resulted in notable enhancements, including reduced neck disability, improved quality of life, and better posture. These outcomes underscore the efficacy of stabilization exercises as a beneficial intervention for individuals dealing with cervical radiculopathy. Furthermore, neural tissue mobilization has proven effective in alleviating pain, enhancing cervical range of motion, and improving functional activity in individuals aged 25 to 40 months with cervicobrachial pain [7].

In the study by Yun et al., both groups exhibited noteworthy distinctions in VAS, NDI scores, and endurance, with significant variances detected between the two groups except for endurance (p < 0.05). Within the intermittent cervical segment traction (ICST) group, considerable variations were noted across all ranges of motion, while in the intermittent cervical total traction (ICTT) group, significant disparities were only observed in extension. Furthermore, significant discrepancies were identified between the two groups (p < 0.05) [8].

In neurodynamics, ICST and ICTT are techniques for treating cervical radiculopathy and neck-related conditions. ICST targets specific cervical spine segments (e.g., C4-C7), applying traction to individual joints or segments to mobilize facet joints and surrounding soft tissue, thereby relieving pressure on individual nerve roots and facet joints. In contrast, ICTT applies traction to the entire cervical spine, mobilizing the vertebral bodies, discs, and facet joints to relieve pressure on multiple nerve roots and the spinal cord, enhancing spinal flexibility and posture. The key difference is that ICST focuses on specific segments and individual nerve roots, while ICTT addresses the entire cervical spine and multiple nerve roots.

Bukhari et al. compared the effectiveness of mechanical and manual traction combined with mobilization and exercise therapy in patients with cervical radiculopathy, finding both approaches significantly improved pain and function, but mechanical traction showed slightly better results in reducing pain and disability [9].

Chiu and Sing evaluated the reliability and validity of assessing cervical range of motion and isometric neck muscle strength, finding high reliability (intraclass correlation coefficients >0.85) and validity for these measurements. Their study supported the use of these metrics for accurately assessing neck function in clinical settings [10].

Rodine and Vernon conducted a systematic review on treating cervical radiculopathy with spinal manipulation, measured using the NDI. They found that spinal manipulation significantly reduced NDI scores, indicating improved neck function and reduced pain, with a majority of studies showing positive outcomes for this treatment method [11].

Stabilization exercise training could be an effective intervention for decreasing pain and improving quality of life and posture in patients with cervical radiculopathy [12]. Neural mobilization may be useful in providing an immediate change in hand grip strength in patients with cervical radiculopathy [13].

The results of this study suggest that the combination of neural tissue mobilization and cervical stabilization exercises may be a valuable addition to the current treatment approaches for patients with unilateral cervical radiculopathy. These interventions could help reduce pain, improve functional status, and increase the cervical range of motion, ultimately leading to better outcomes for patients with this condition [14].

In a randomized, double-blinded, placebo-controlled clinical trial by Savva et al., at the four-week follow-up, cervical traction in combination with neural mobilization resulted in improved outcomes in pain, function, and disability in patients with cervical radiculopathy [15].

However, this study had some limitations, including a small sample size and the lack of a long-term follow-up. Future research with larger sample sizes and longer follow-up periods is needed to further investigate the effectiveness of these interventions in patients with cervical radiculopathy.

## Conclusions

Cervical stabilization exercises among unilateral cervical radiculopathy patients have shown promise in effectively reducing pain, enhancing cervical range of motion, and improving the craniovertebral angle while mitigating neck function disability in unilateral cervical radiculopathy individuals, surpassing the outcomes of conventional stretching and strengthening exercises targeting neck, shoulder, and scapular muscles.

Future studies may expand to include bilateral cervical radiculopathy patients to evaluate the applicability of neural tissue mobilization and explore its effectiveness for acute cases. Long-term follow-ups could offer insights into treatment sustainability, while diverse interventions might enhance the quality of life for cervical radiculopathy patients.

## References

[1] Radhakrishnan K, Litchy WJ, O'Fallon WM, Kurland LT (1994). Epidemiology of cervical radiculopathy. A population-based study from Rochester, Minnesota, 1976 through 1990. Brain.

[2] Carette S, Fehlings MG (2005). Clinical practice. Cervical radiculopathy. N Engl J Med.

[3] Kim DG, Chung SH, Jung HB (2017). The effects of neural mobilization on cervical radiculopathy patients' pain, disability, ROM, and deep flexor endurance. J Back Musculoskelet Rehabil.

[4] Basson A, Olivier B, Ellis R, Coppieters M, Stewart A, Mudzi W (2017). The effectiveness of neural mobilization for neuromusculoskeletal conditions: a systematic review and meta-analysis. J Orthop Sports Phys Ther.

[5] Srinivasulu M, Chunduri D (2021). Comparing Mulligan mobilization and neural mobilization effects in patients with cervical radiculopathy. RGUHS J Physiother.

[6] Kuo YL, Lee TH, Tsai YJ (2020). Evaluation of a cervical stabilization exercise program for pain, disability, and physical impairments in university violinists with nonspecific neck pain. Int J Environ Res Public Health.

[7] Ganesh Ganesh, Sonam B (2019). Effectiveness of neural tissue mobilization in the management of cervicobrachial pain in subjects between age group 25-40 years. Indian J Physiother Occup Ther.

[8] Yun YH, Lee BK, Yi JH, Seo DK (2020). Effect of nerve mobilization with intermittent cervical segment traction on pain, range of motion, endurance, and disability of cervical radiculopathy. J Phys Ther Rehabil Sci.

[9] Bukhari SR, Shakil-Ur-Rehman S, Ahmad S, Naeem A (2016). Comparison between effectiveness of mechanical and manual traction combined with mobilization and exercise therapy in patients with cervical radiculopathy. Pak J Med Sci.

[10] Chiu TT, Sing KL (2002). Evaluation of cervical range of motion and isometric neck muscle strength: reliability and validity. Clin Rehabil.

[11] Rodine RJ, Vernon H (2012). Cervical radiculopathy: a systematic review on treatment by spinal manipulation and measurement with the Neck Disability Index. J Can Chiropr Assoc.

[12] Akkan H, Gelecek N (2018). The effect of stabilization exercise training on pain and functional status in patients with cervical radiculopathy. J Back Musculoskelet Rehabil.

[13] Nair R, Holla S, Rajadhyaksha S (2017). Effect of neural tissue mobilization on grip strength in patients with cervical
radiculopathy. J Soc Indian Physiother.

[14] Pawaria S, Sudan DS, Kalra S, Yadav J (2019). Effectiveness of cervical stabilization exercises with feedback on respiratory status in chronic neck pain patients with forward head posture. Int J Physiother.

[15] Savva C, Korakakis V, Efstathiou M, Karagiannis C (2021). Cervical traction combined with neural mobilization for patients with cervical radiculopathy: a randomized controlled trial. J Bodyw Mov Ther.

